# Functional outcome after one-stage flap reconstruction of the hypopharynx following tumor ablation

**DOI:** 10.1007/s00405-016-4279-8

**Published:** 2016-08-26

**Authors:** Talisa D. van Brederode, Gyorgy B. Halmos, Martin W. Stenekes

**Affiliations:** 1 Department of Plastic Surgery, University of Groningen, University Medical Center, Groningen, Postbus 30.001, 9700 RB Groningen, The Netherlands; 2 Department of Otorhinolaryngology, Head and Neck Surgery, University of Groningen, University Medical Center Groningen, Groningen, The Netherlands

**Keywords:** Reconstruction, Free flaps, Pectoralis major, Hypopharynx, Interposition flap

## Abstract

The main objective of this study was to evaluate functional outcome in terms of food passage of the three different reconstruction techniques that are currently most often used for hypopharyngeal reconstruction in our institution. A retrospective observational database research was conducted of all patients that underwent hypopharyngeal reconstruction for carcinoma of the hypopharynx or larynx from 1992 until 2014 in the University Medical Center Groningen. The following techniques were most commonly used and therefore analyzed: the pedicled pectoralis major flap, the radial forearm free flap and the anterolateral thigh free flap. Our primary outcome food passage was measured after 1 year and classified in gastric tube fed, fluids, semisolid or solid. Complications were registered according to the Clavien Dindo classification in five different grades. Comorbidity was scored using the Adult Comorbidity Evaluation Index. 58 patients were included. 51 patients survived one year follow up, 25 % returned to a solid diet, 40 % returned to a semi-solid diet and 20 % remained feeding tube dependent. Overall flap success rate was 88 and 35 % developed a pharyngocutaneous fistula. Multivariable ordinal regression showed that reconstruction with free flaps, a near-circumferential surgical defect, a higher body mass index and no comorbidity showed significantly better functional outcomes in the food passage. For recipient site complications, both free flaps and a shorter surgery time resulted in less severe complications. This study shows that the use of free flaps is superior to the use of the pectoralis major flap, and that it should therefore be reserved as a second choice.

## Introduction

Surgery of advanced laryngeal and hypopharyngeal carcinoma may include partial or total laryngectomy with or without partial or total pharyngectomy. This will potentially leave large, sometimes circumferential defects which require complex reconstruction [1, 2].

There are many factors that contribute to the choice of a certain reconstruction technique. For instance, the size and level of the defect, patient’s general medical health and wishes, the surgeon’s experience and preference of different techniques are all factors to be taken into consideration. The transferred tissue needs to withstand high dose radiation therapy (usually adjuvant radiotherapy is required), not bulky and pliable so it can cover a circumferential defect and preferably not be too close to the neck so that two teams can operate simultaneously [3].

Many reconstruction techniques have been developed and successfully used. Myocutaneous pedicled flaps (pectoralis major, deltopectoral, latissimus dorsi), visceral transposition (jejunal autograft, colon autograft, gastric pull-up), free fasciocutaneous flaps (anterolateral thigh, radial forearm, scapular) and more have been described in literature [4]. As regarding to complication rates and functionally in swallowing and speech, each technique has its advantages and limitations.

The surgical defect that arises in the hypopharynx after tumor ablation can be circumferential, near-circumferential or partial (the latter is left out of consideration in this study). Sometimes it is oncologically safe to leave a mucosal strip of the posterior pharyngeal wall intact. This means that the reconstruction flap is sutured to the posterior pharyngeal wall, creating two additional vertical suture lines. In theory this could be a potential point of weakness, leading to dehiscence of the flap and pharyngocutaneous fistulas. On the other hand, some experts suggest that a near-circumferential defect creates less long-term strictures [1].

In our institution we generally use three types of hypopharyngeal reconstruction, namely the anterolateral thigh free flap (ALTFF), the radial forearm free flap (RFFF) and the pedicled pectoralis major myocutaneous flap (PMMF). The PMMF is often too bulky, making it aesthetically and functionally non-satisfying. Therefore, the PMMF is preserved as a second choice in our institution, for high risk patients in whom a free vascularized fasciocutaneous flap has failed or is considered too risky. The RFFF and ALTFF are both considered to have good results, although the ALTFF cannot be used in obese patients.

Currently there is no consensus on the primary preference for hypopharyngeal reconstruction. The objective of the present study is to evaluate functional outcome viz. voice rehabilitation and food passage of the three different reconstruction techniques that are currently used in this institute.

## Materials and methods

In this retrospective observational database study, clinical records of all patients that underwent hypopharyngeal reconstruction for carcinoma of the hypopharynx or larynx between 1992 and 2014 in the University Medical Centre Groningen were collected. These defects were reconstructed using RFFF, ALTFF or PMMF.

### Clinical variables

Patient and treatment information was obtained from electronic patient dossiers. The following variables were included: age, sex, tumor site, cancer stage, preoperative body mass index (BMI), postoperative BMI at discharge, the American Society of Anesthesiologists (ASA)-score, pre- and postoperative therapy (such as chemotherapy or radiotherapy), any history of malignancy, length of the surgery, type of reconstruction, surgical defect, the number of hospital days and postoperative complains such as dysphagia, reflux and aspiration. The Adult Comorbidity Evaluation 27 (ACE-27) index was used to score preoperative comorbidity [5].

### Outcome measures

Functionality in food passage after full recovery was classified in the following outcome categories: gastric tube fed fluids, fluids, semisolid (pureed soft food) or solid. This information was preferably retrieved from a dietician report, approximately one year postoperatively.

Speech function after reconstruction was also evaluated. The type of speech used was classified in: electrolarynx, esophageal speech or tracheoesophageal puncture and prosthesis. The quality and patient satisfaction of speech were also evaluated.

Complications were registered according to the Clavien Dindo classification in five different grades [6, 7]. These complications were classified as related to the recipient site, donor site and medical complications [8, 9]. Clinically relevant complications were also listed separately, such as flap necrosis and pharyngocutaneous fistulas.

### Statistical analysis

For statistical analysis IBM SPSS Statistics 22.0 was used [10]. Descriptive statistics was used to display patient characteristics, treatment details and functional outcome. To evaluate significant factors that affect the food passage and recipient site complications, multivariable ordinal regression was used.

There are probably more factors contributing to both the choice of flap reconstruction and the functional outcome food passage. Based on univariable ordinal regression (with *α* = 15 % [11]), expert opinion and literature, five variables were identified for multivariable ordinal regression analysis. These variables were: type of reconstruction (PMMF, ALTFF, RFFF), surgical defect (circumferential or near-circumferential), ACE-27 (grade III, grade II, grade I, none), postoperative radiotherapy (yes or no) and preoperative BMI (continuous variable).

Recipient site complications is also an ordinal variable, classified in grades of severity from no complication at all to grade V, meaning death. In all likelihood, more factors than just the type of reconstruction used will have an impact on recipient site complications. In a similar way as described above, five variables were selected to participate in the multivariable ordinal regression model. These variables were: type of reconstruction (PMMF, ALTFF, RFFF), surgical defect (circumferential or near-circumferential), ACE-27 (grade III, grade II, grade I, none), length of the surgery (scale variable, defined in minutes) and preoperative radiotherapy (yes or no).

## Results

### Demographics

In total 58 patients were included in this study. The median age was 62 years, and most patients were men (male:female ratio: 4.27:1). Median BMI was 23.3 kg/m^2^. Most patients had mild comorbidity according to the ACE-27 index. Over half of the patients have had previous radiotherapy in the head and neck area. 18 patients had already undergone total laryngectomy, after which the cancer had recurred or a second primary tumor developed, needing further resection and therefore reconstruction. For further demographic details, see Table 1.

**Table 1:** Preoperative characteristics

*N* = 58	*n* (%)	Median (range)
Age (years)		62 (43–88)
Sex		
Male	47 (81.0)	
Female	11 (19.0)	
BMI (kg/m^2^)		23.3 (14.4–37.1)
ASA-classification		
ASA 1	3 (5.2)	
ASA 2	22 (37.9)	
ASA 3	26 (44.8)	
ASA 4	2 (3.4)	
N/A	5 (8.6)	
Pre-operative co-morbidity (ACE-27)		
None	16 (27.6)	
Mild	28 (48.3)	
Moderate	13 (22.4)	
Severe	1 (1.7)	
Primary tumor site		
Hypopharynx	39 (67.2)	
Larynx	19 (32.8)	
Cancer stage		
Stage III	3 (5.2)	
Stage IV	24 (41.4)	
Recurrent cancer	27 (46.6)	
N/A	4 (6.8)	
History of malignancy	21 (36.2)	
Head and Neck	18 (31.0)	
Lung	2 (3.4)	
Other	4 (6.9)	
Previous treatment		
None	22 (37.9)	
(Chemo)radiation	36 (62.1)	
Total laryngectomy	18 (31.0)	

### Surgical outcome

There were almost as many circumferential as near-circumferential hypopharyngeal defects (respectively, 32 and 26). In total 16 pectoralis major flaps, 11 anterolateral thigh flaps and 31 radial forearm flaps were used. Median length of laryngectomy and reconstruction was 657 minutes, ranging from 267 until 1128 minutes. Three patients died within one month after surgery, which leads to an early mortality rate of 5.2 % in this population. Two of these patients died because of medical complications after major surgery (e.g. congestive heart failure and/or untreatable electrolyte disturbances leading to multiple organ failure). One patient died because of major untreatable recipient site complication. Patients were hospitalized for an average of 27 days. Over half of the patients received additional treatment in the form of radiotherapy or chemoradiation. For more treatment details, see Table 2.

**Table 2:** Treatment details

*N* = 58	*n* (%)	Median (range)
Surgical hypopharynx defect		
Circumferential	32 (55.2)	
Near-circumferential	26 (44.8)	
Type of reconstruction		
Pectoralis Major	16 (27.6)	
Circumferential	4 (6.9)	
Near-circumferential	12 (20.7)	
Anterolateral thigh	11 (19.0)	
Circumferential	10 (17.2)	
Near-circumferential	1 (1.7)	
Radial forearm	31 (53.4)	
Circumferential	18 (31.0)	
Near-circumferential	13 (22.4)	
Length of surgery (minutes)^a^		657 (267–1128)
Hospital stay (days)		27 (16–137)
Postoperative treatment		
None	22 (37.9)	
Chemo(radiation)	36 (62.1)	

^a^Length of surgery includes total laryngectomy and reconstruction

### Functional outcome

52 patients survived the one year follow up period, but one was excluded for functional outcome data because a jejunum flap was used to restore the pharynx after total necrosis of the original flap. Of these 51 patients, about 80 % was able to be self-sufficient in oral nutrition. Almost 20 % was not able to swallow at all, and was therefore feeding tube dependent. Median BMI one year postoperative was 23.2 kg/m^2^, 0.1 kg/m^2^ lower than preoperative.

Strictures that needed endoscopic dilatation occurred in 38 % of patients. Patients required a median of four dilatations each in their follow up period, ranging from 1 up to 70. Most patients needed two till four dilatations with good functional results. One patient required a monthly dilatation but kept having good results, so that he has had a total of 70 dilatations now. The number of dilatations was not dependent on years of follow up.

About 75 % of the patients were able to communicate with the tracheoesophageal puncture (TEP) and prosthesis, and about 50 % of patients considered their speech as medium to good. Over 50 % of patients were satisfied with the reconstruction results, with mild complaints. 37 % of the patients complained of dysphagia postoperatively. For more details in functional outcome, see Table 3.

**Table 3:** Functional outcome

*n* = 51	*n* (%)	Median (range)
BMI (kg/m^2^)		23.2 (16.3–35.9)
Food passage		
Solid	14 (27.5)	
Semisolid	21 (41.2)	
Fluids	6 (11.8)	
Gastric tube/PEG	10 (19.6)	
Postoperative complaints		
Dysphagia	19 (37.5)	
Reflux	3 (5.9)	
Aspiration	4 (7.8)	
Speech type		
Electrolarynx	3 (5.9)	
Esophageal speech	5 (9.8)	
TEP and prosthesis	39 (76.5)	
No speech	4 (7.8)	
Speech quality^a^		
Good	7 (13.7)	
Medium	20 (39.2)	
Bad	21 (41.2)	
Patient satisfaction^a^		
Very satisfied	4 (7.8)	
Mild complaints	25 (49.0)	
Major complaints	21 (41.2)	

^a^Not all patients recorded speech quality and overall satisfaction

Food passage was compared between the different types of reconstruction. The pectoralis major pedicled flap had the highest percentage of feeding tube dependent patients (35.3 %), whereas only 11.8 % of free flaps were tube dependent one year postoperatively. In the pectoralis major pedicled group, only 17.6 % of patients regained a fully solid diet, while in the free flap group this was 32.4 % (Table 4).

**Table 4:** Food passage after one year in different reconstruction groups

*N* = 51	Solid *n* (%)	Semi-solid *n* (%)	Fluids *n* (%)	GT/PEG *n* (%)	Total *n* (%)
Pedicled flaps					
Pectoralis major	3 (17.6)	6 (35.3)	2 (11.8)	6 (35.3)	17 (100.0)
Free flaps (total)	11 (32.4)	15 (44.1)	4 (11.8)	4 (11.8)	34 (100.0)
Anterolateral thigh	2 (22.2)	4 (44.4)	2 (22.2)	1 (11.1)	9 (100.0)
Radial forearm	9 (36.0)	11 (44.0)	2 (8.0)	3 (4.0)	25 (100.0)

### Complications

No complication was registered in only 10 % of patients during the postoperative period. 65 % suffered some sort of recipient site complication, ranging from partial flap necrosis (5.2 %), wound dehiscence (22.4 %), wound infections (24.1 %), pharyngocutaneous fistulas (36.2 %) and complete flap necrosis (12.1 %). When we split up complete flap necrosis for each reconstruction type, the pectoralis major has a success rate of 87.5 % (two flaps died because of anatomical vascular variation), the radial forearm 87.0 % and the anterolateral thigh 90.9 %. Combined free flaps have a total success rate of 88.1 %.

15.5 % of patients had a donor site complication, where 6.9 % needed re-intervention in the operating room. 31.0 % had some sort of medical complication, varying from electrolyte disturbances to respiratory failure. Two patients suffered from major medical complications, leading to death. For more details, see Table 5.

**Table 5:** Total amount of complications, categorized according to recipient site, donor site and medical complications

*N* = 58	Recipient site *n* (%)	Donor site *n* (%)	Medical *n* (%)
None	20 (34.5)	49 (84.5)	40 (69.0)
Grade I	7 (12.1)	4 (6.9)	5 (8.6)
Grade II	6 (10.3)	0	10 (7.2)
Grade III	24 (41.4)	5(8.6)	0
Grade IV	0	0	1 (1.7)
Grade V	1 (1.7)	0	2 (3.4)

Twenty-one patients (36.2 %) developed a pharyngocutaneous fistula in the postoperative period. More than half of these fistulas (11 patients, 52.4 %) were successfully treated with conservative treatment. Nine patients (42.9 %) needed flap reconstruction with a pectoralis major pedicled flap to resolve the fistula. One patient (4.8 %) could suffice with sutures for closure of the fistula (Table 6). Comparing the occurrence of fistula versus the type of surgical defect, we found no significant difference in the occurrence of fistulas (Chi-square, *p* = 0.664).

**Table 6:** Pharyngocutaneous fistula per surgical defect

	Circumferential *n* = 32	Near-circumferential *n* = 26	Total *n* = 58
Pharyngocutaneous fistulas	12 (37.5)	9 (34.6)	21 (36.2)
Intervention			
Conservative treatment	7 (58.3)	4 (44.4)	11 (52.4)
Sutures	1 (8.3)	0	1 (4.8)
Flap surgery	4 (33.3)	5 (55.5)	9 (42.9)

Besides pharyngocutaneous fistulas, there were also a few problematic tracheoesophageal fistulas. These are iatrogenic fistulas, designed to hold the speech prosthesis. In total there were 53 out of 58 patients who received a TEP during, or shortly after the reconstruction surgery. Of those patients, nine (17.0 %) had a complication with the TEP. Four of these patients (7.5 %) could suffice with conservative treatment, another four needed flap surgery and one patient (1.8 %) needed additional sutures.

### Multivariable ordinal regression analysis

#### Food passage

Multivariable ordinal regression analysis showed a significant difference between the pectoralis major flap versus radial forearm flap (*p* = 0.005) and the pectoralis major versus anterolateral thigh flap (*p* = 0.006), in favor of both free flaps. When data was transformed to compare the radial forearm and anterolateral thigh flap, no significant result was found (*p* = 0.365). Other significant outcomes were: circumferential surgical defect versus near-circumferential surgical defect (*p* = 0.004), in favor of the near-circumferential surgical defect. Preoperative BMI (*p* = 0.016) was also found to be significant, with an odds ratio below 1, meaning with each point increase in BMI a higher cumulative score is more likely, suggesting a better functional outcome when BMI is high. No comorbidity versus grade II comorbidity (ACE-27 index) (*p* = 0.041) was also significant, in favor of no comorbidity. Postoperative radiation therapy did not appear to be significant in affecting the food passage (*p* = 0.267). Multivariate data with odds ratios are shown in Table 7.

**Table 7:** Multivariable ordinal regression for food passage after full recovery

Variable	Odds ratio (95 % confidence interval)	*p* value
Type of reconstruction		
Pectoralis major	1 (ref)	–
Radial forearm	0.11 (0.02–0.51)	0.005
Anterolateral thigh	0.05 (0.01–0.42)	0.006
Surgical defect		
Near-circumferential	1 (ref)	–
Circumferential	8.58 (2.00–36.78)	0.004
BMI preoperative	0.82 (0.69–0.96)	0.016
Postoperative radiotherapy		
Yes	1 (ref)	–
No	0.44 (0.10–1.89)	0.267
ACE-27		
None	1 (ref)	–
Grade I	4.14 (0.92–18.60)	0.064
Grade II	8.96 (1.09–73.55)	0.041
Grade III	23.59 (0.25–2257.47)	0.174

*p* < 0.05 was considered statistically significant

#### Complications

In multivariable analysis for recipient site complications, both free flaps show significant less recipient site complications (radial forearm *p* = 0.021, anterolateral thigh *p* = 0.013). A longer duration of the surgery time was also found to be a significant predictor of recipient site complications (*p* = 0.026). However, in this model the type of surgical defect, preoperative radiotherapy and comorbidity were found not to be significant factors in predicting recipient site complications. Multivariate analysis and odds ratio are also shown in Table 8. When transforming the data to compare the anterolateral thigh flap with the radial forearm flap, no significant result was found (*p* = 0.209).

**Table 8:** Multivariable ordinal regression for recipient site complications

Variable	Odds ratio (95 % confidence interval)	*p* value
Type of reconstruction		
Pectoralis major	1 (ref)	–
Anterolateral thigh	0.04 (0.01–0.52)	0.013
Radial forearm	0.08 (0.01–0.68)	0.021
Surgical defect		
Circumferential	1 (ref)	–
Near-circumferential	1.82 (0.48–6.85)	0.375
Preoperative radiotherapy		
Yes	1 (ref)	–
No	1.05 (0.26–4.25)	0.943
Length of surgery	1.01 (1.00–1.01)	0.026
ACE-27		
None	1 (ref)	–
Grade I	1.23 (0.27–5.52)	0.791
Grade II	1.23 (0.18–8.67)	0.832
Grade III	1.29 (0.01–573.07)	0.935

*p* < 0.05 was considered statistically significant

## Discussion

This study shows that hypopharyngeal reconstruction with a free radial forearm flap and free anterolateral thigh flap leads to a significantly better functional outcome, and less recipient site complication than the pectoralis major pedicled flap.

To our knowledge we are the first to use multivariable ordinal regression analysis to compare different types of reconstruction on the outcomes food passage and recipient site complications using the standardized Clavien Dindo classification system [6]. As anticipated, surgical defect (circumferential or near-circumferential) found to be a significant factor to determinate postoperative food passage. A near-circumferential defect resulted in better food passage. However, in contrast to the expectations, our results also show that the type of surgical defect does not make a difference in the formation of pharyngocutaneous fistulas. Creating two extra vertical suture lines on the posterior pharyngeal wall, does not appear to be a significant contributor to more recipient site complications, nor the formation of fistulas.

Multiple studies have demonstrated that free flaps such as the radial forearm free flap and anterolateral thigh free flap have fewer complications and the same or even better functional results regarding swallowing and speech rehabilitation when compared to a free jejunal flap [12–15]. Nevertheless, there are many articles considering the free jejunal flap as the ‘golden standard’ for circumferential defects. Proponents of the jejunal flap claim it has some sort of peristalsis; it is naturally tubularized and easily fits to the cranial and caudal end of the pharynx. Downsides of the jejunal flap are that it requires abdominal surgery with its additional risks, and the re-vascularized jejunal flap does not seem to withstand high dose radiotherapy. Also, jejunal flaps provide poor speech rehabilitation, as the speech tends to stay ‘wet’ [16, 17].

We found that 11 % of the free flap group and 35 % of the pedicled group remained dependent on a gastric tube or PEG. This is comparable with another retrospective study, including 94 patients [12], which showed 26 % of patients in the pedicled group depending on a gastric tube or PEG, compared to 14 % in the free flap group. Not being able to provide oneself with oral nutrition has an enormous impact on quality of life [18]. The differences between free flap and pedicled flap are even more striking in the study by Mura et al. [12]; in their free flap group 80 % of patients returned to a normal unrestricted diet, compared to 0 % in the pedicled flap group. In the pedicled flap group most patients (74 %) remained dependent on a semi-solid diet.

In a retrospective analysis by Benazzo et al. [13], 75 % of free flap patients resumed a normal diet, whereas 25 % was dependent on a soft diet. This appears inconsistent with our results, where 30 % of patients with hypopharyngeal reconstructions with a free flap resumed a solid diet; however, in this study the term “soft diet” was not specified. In case the authors meant solid and semi-solid combined for “a normal diet”, our results are roughly comparable.

In a retrospective analysis of 136 patients by Van der Putten et al. [1], different types of hypopharyngeal reconstruction were compared. They showed that 82 % retained a fully oral diet without the need of gastric tube or PEG, which is also similar to our results (combined solid, semi-solid and fluids is 81.4 % in our results). Pharyngocutaneous fistula rate in this study was 35 %, which is also comparable with our results. In contrast to our findings, only 38 % of laryngectomized patients had functional voice prosthesis after one year, compared to 75 % in our study group.

In all other studies discussed above, it must be mentioned that jejunal transposition flaps are also included in the free flap groups, a reconstruction technique we only use with high exception in our institution. The way pharyngocutaneous fistulas are reported in literature is inconsistent. Some studies only register pharyngocutaneous fistulas where intervention is required as a complication. In contrast to this, we reported fistulas as a leakage on barium swallowing, independently of the intervention (conservative, sutures or flap surgery) that was required. A meta-analysis, published in 2008 also struggled with these inconsistencies [19]. In this meta-analysis of 20 papers about fasciocutaneous free flaps used for pharyngoesophageal reconstruction, a combined fistula rate of 13 %, and a stricture rate of 16 % were found. This is far less than the 35 % fistula rate and 37 % stricture rate that we found. Overrating our fistula rate and inconsistent reporting in literature might be an explanation for the difference found.

Our overall flap failure rate was 12 %, with a slightly higher flap failure rate in the pedicled flap group than the free flap group (13 versus 11 %). When we compare this with other studies, combined flap failure rates varies from 0 to 9.5 % [1, 13, 17, 19–21]. Pedicled pectoralis major often has a failure rate of 0 %, because there is no need for microvascular anastomosis [12, 13]. Instead, we found a flap failure in two patients, both due to abnormal vascular anatomy. In a few studies discussed above, patients were fitter preoperatively, had less advanced disease and less preoperative radiotherapy. This might explain the difference in flap failure rate. In our institution we have a high percentage of salvage surgery leading to impaired wound healing, and higher percentage of complications such as pharyngocutaneous fistulas or flap failure [22–24]. Despite our knowledge and generally accepted consensus that radiotherapy has a negative influence on wound healing, in our multivariable analysis this did not seem to make a significant difference. This finding is inconsistent with literature, but might be explained because more than half of our patients needed pre- and/or post-operative radiotherapy, so that this variable no longer makes a significant difference.

A prospective study should be set up to rule out selection bias in this study group. Such a study should analyze additional data, such as suturing techniques (interrupted or continuous, single loose or mattress), neck dissections (uni- or bilateral) and the value of an additional pectoralis major flap. The latter is suggested by some surgeons and may protect the free flap, cover exposed great vessels, minimize the risk of wound dehiscence and fistula formation [25, 26]. Besides that, a more detailed dietician report must be kept at fixed intervals postoperatively of the patient’s ability to swallow, and possible complaints such as dysphagia, aspiration and reflux. Speech function and patient satisfaction should be tested objectively, for instance with the Mendelsohn’s scale [27] and validated quality of life questionnaires [28] at fixed intervals.

Despite the fact that flap reconstruction of the hypopharynx after pharyngolaryngectomy remains challenging and a complex problem for head and neck surgeons, this study shows that in the majority of cases satisfactory functional results in terms of swallowing and speech rehabilitation can be achieved.

Our data shows that the pectoralis major group is more prone to get higher grades of recipient site complications, and a worse functional outcome. Both anterolateral thigh- and radial forearm free flap show significant better functional outcome in swallowing, and less recipient site complications. Based on these results, we conclude that free flap reconstruction of hypopharyngeal defects must remain the first choice whenever patients are fit enough. A pedicled pectoralis major flap should be reserved for cases where a free flap isn’t safe or possible. A prospective, most preferably multicentre study is recommended to eliminate selection bias of patients.
